# Recognition of Colon Polyps (Tubular Adenoma, Villous Adenoma) and Normal Colon Epithelium Histomorphology with Transfer Learning

**DOI:** 10.5152/eurasianjmed.2024.23130

**Published:** 2024-02-01

**Authors:** Ibrahim Karabulut, Rabia Selen, Mete Yaganoglu, Sevilay Ozmen

**Affiliations:** 1 Atatürk University Faculty of Medicine, Erzurum, Turkey; 2 Department of Computer Engineering, Atatürk University Faculty of Engineering, Erzurum, Turkey; 3 Department of Pathology, Atatürk University Faculty of Medicine, Erzurum, Turkey

**Keywords:** Machine learning, transfer learning, colon, normal epithelium, tubular adenoma, villous adenoma

## Abstract

**Background:**

The use of artificial intelligence technology in medicine, which is remarkable with its increasing use in many areas recently, has allowed for rapid developments. This technology quantitatively solves many problems in medicine, such as increased workload, delayed diagnosis, and treatment processes. It has been seen in the literature that this technology, which has a wide range of applications in medicine, also has a place in medical pathology. The main purpose of this study is to provide a histomorphological classification of colon polyps in the medical pathology department with high accuracy in a short time. Besides accelerating the diagnosis and treatment process, it is desired to facilitate the workload of the pathology department.

**Methods:**

This study is based on the recognition of colon preparation images that come to the pathology department by using the image recognition techniques of artificial intelligence. VGG19, DenseNet201, and EfficientNetB7 models, which are convolutional neural networks (CNN) models, were used. A model based on a concatenation ensemble of 3 CNN architectures was also used in this study.

**Results:**

Within the scope of the study, a total of 515 preparation images, including normal epithelium, tubular adenoma and villous adenomas of the colon, were procured as data and introduced to artificial intelligence, and the diagnoses of these preparations were estimated histomorphologically by artificial intelligence.

**Conclusion:**

As a result of the study, an accuracy rate of 94.17% was achieved.

**Main Points**

- This application has used VGG19+ DenseNet201+ EfficientNetB7 models.
- This application diagnosed preparations with 94.17% accuracy.
- In this application, more accurate diagnosis rate was achieved with less data.
- With this application, the data were introduced to AI in 13 minutes and 34 seconds.

## Introduction

Medical pathology is the branch of science that diagnoses the disease by histomorphologically recognizing the preparations and tissue samples from clinical departments through the microscope. Since this recognition process has some problems such as the large number of these samples coming from the clinical departments and the insufficient quality of the preparation image prolonging the process of diagnosing the disease, delays may occur in the treatment. Also, the increased workload in the pathology department complicates the developments in this department and the training of qualified personnel, especially in training and research hospitals. In addition, the shortage of physicians (personnel) in many places is becoming significant. In a study by Ashish et al in India in 2021, it was shown that the ratio of radiologists to patients poses a significant challenge in diagnosing the diseases.^1^

Artificial intelligence has been used in clinical decision support systems since the 1970s.^1^ Artificial intelligence has also made a place for itself in many fields of medicine. In the pulmonary function test evaluation performed by Tapalovic et al^2^ in the field of pulmonology, pulmonologists correctly diagnosed 24%-62% of cases, while artificial intelligence correctly diagnosed 82% of cases. In another study, an artificial intelligence application called convolutional neural networks (CNN) that works with an artificial neural network with a learning feature was used. The performance of 58 dermatologists from different countries and the performance of CNN were compared to each other in diagnosis of malignant melanoma, a type of skin cancer. In this study, physicians made correct diagnoses with a rate of 86.6% while CNN was more successful with a rate of 95% correct diagnoses than all the physicians participating in the research.^3^ In a study by Teramato et al^4^ on chest diseases, artificial intelligence recognized the histological subtypes of lung cancer with an accuracy rate of 60%-89%. There are studies on the use of artificial intelligence in many medical fields, especially in gastroenterology, orthopedics, traumatology, dermatology, and pulmonology. In the study conducted in the field of ophthalmology for the diagnosis of diabetic retinopathy, 75 137 fundus images were used, and 94% sensitivity and 98% specificity were obtained.^5^ In another study, 17 574 endoscopy images were diagnosed with an accuracy of 88.6%.^6^

Artificial intelligence is also used in tumor diagnosis, classification, staging, grading, and prognosis prediction. In the study by Tomita et al^7^ in 2019, it was seen that artificial intelligence classified gastric lesions as normal, dysplastic epithelium, and cancer with an accuracy rate of 85.6%. In addition, artificial intelligence is used in the detection of tumor metastases. Deep learning algorithms have been used for primary lymph node staging, especially in patients with abdominopelvic malignancies.^8^

Colon cancer is the third most common cancer in the world. Premalignant polyps seen in the colon are associated with cancer prevalence. Especially as the rate of a villous component in the adenomatous polyp increases, the risk of developing cancer in that polyp increases.^9^ Early diagnosis and removal of adenomatous polyps are reducing cancer-related morbidity and mortality.^10^ Therefore, the correct diagnosis and treatment of premalignant polyps of the colon is important. Nasir et al used the ResNet-18 model to classify colon polyps with an accuracy rate of 87%.^11^ In the study of Wei et al^12^ in 2019 to identify colon polyps, the ResNet model was used, and an accuracy rate of 93.5% was achieved with 1182 preparation images. An overall accuracy rate of 93% was obtained from 2074 colon polyp images that Korbar et al^13^ taught to the ResNet model in 2017.

Misdiagnosis of pathology materials is also inevitable in this process. For example, in a total of 90 000 frozen examinations conducted in 461 centers with the sponsorship of the College of American Pathologists (CAP), 98.58% accuracy was achieved. 67.8% of the unidentified cases were insufficient to detect neoplasia. The reasons for inaccuracy are incorrect evaluation (31.8%), sampling error (31.4%), and the presence of tissue in the frozen section while it was not seen in the permanent section (30%). As demonstrated in this study, incorrect assessment is an important cause of unidentified cases.^14^ In addition, the classification system of colon cancer neoplasms is still a matter of debate in the world. Vienna and Japanese classification systems are widely used for classification, but tumor invasion is a diagnostic feature for carcinoma in the Vienna system, whereas the nuclear characteristics of cells are more diagnostic in the Japanese system.^15,16^

In a study by Nasir et al in 2021, artificial intelligence increased the correct diagnosis rate by 21.3% in 1500 images of colon polyps diagnosed by 15 pathologists.^17^ In this study, the normal epithelium, tubular and villous adenomas of the colon were visually introduced to the artificial intelligence, and artificial intelligence made the histomorphological classification. An attempt was made to reduce the error rate of the diagnosis made by artificial intelligence, and an accuracy rate of 94.17% was obtained with a sensitivity of 95.12% for the normal colon epithelium.

The main purpose of this study is to minimize the diagnostic process in the pathology department. In addition, it reduces the rate of misdiagnosis with its high accuracy rate. The results of the current project demonstrate the potential to provide practical support to pathologists. The contribution of the study to the literature supports that the accuracy rate can be kept high depending on the machine learning model to be used, even if the data size is limited.

## Material and Methods

**Figure 1.**
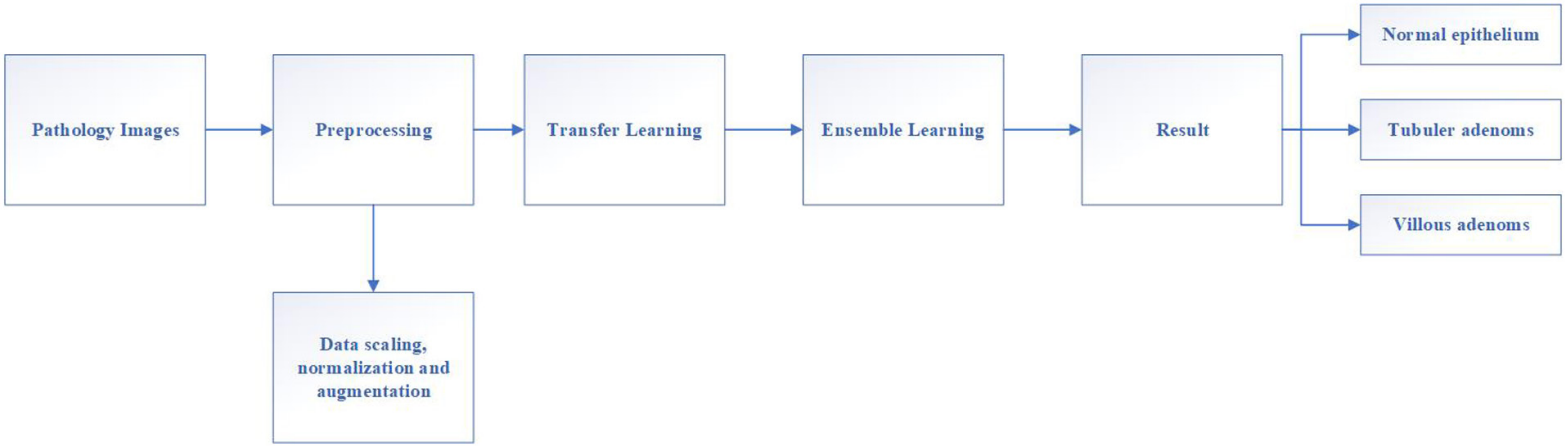
Workflow of the proposed method.

In this study, colon preparation images sent to Ataturk University Faculty of Medicine, Department of Pathology between January 2020 and April 2022 were used. All manipulations and procedures were carried out according to the national guidelines adopted by the relevant ethics council. The study was approved by the Clinical Research Ethics Council of Ataturk University (B.30.2.ATA.0.01.00/398 date: 28.04.2022). Informed consent could not be obtained from the patients as the study was a retrospective study. In this project, 3 classes were tried to be estimated. These are normal epithelium, tubular adenoma, and villous adenoma. All cases were included in this study without being selective (age, gender). The flow chart of the study is seen in Figure 1.

In the study, a total of 515 images were obtained that consisting of 290 images for the normal epithelium of the colon, 102 for tubular adenomas and 123 for villous adenomas. Since there may be noise and low contrast in the obtained images, these images were first subjected to the image preprocessing stage.

**Figure 2.**
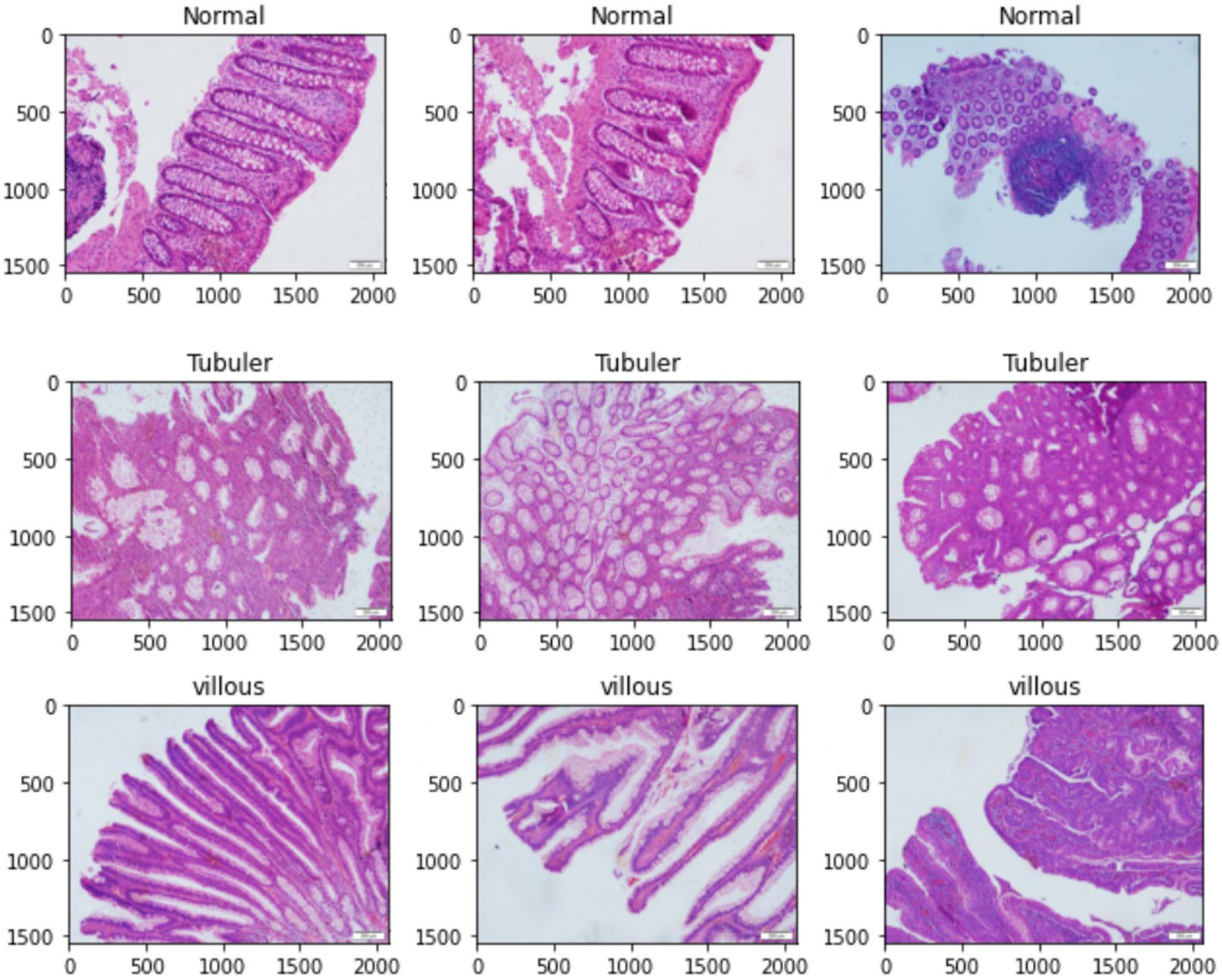
The outlook of the data.

The dataset is categorized into 2 main folders (train and test) and 3 subfolders (normal epithelium, tubular adenomas, villous adenomas) in both of them. The dataset contains a total of 515 images, and the test data include 20% of the total images. The outlook of the data is as seen in Figure 2.

### Image Preprocessing

Image processing is a method in which different algorithms are applied to extract important information from images. It can be defined as a method of transforming an image from one form to another. New images are obtained with image processing by applying various operations to digital images. Operations such as sharpening, increasing the contrast, clarifying some details, softening the image and sharpening the edges are performed on the image with image preprocessing methods. 

Different preprocessing methods to be carried out on the images ensure the optimization of the training process by increasing the quality of the input images. Image filtering, which is defined as an image processing application applied to highlight or suppress some details in digital images, identify edges and reduce noise and negative effects in the image. It also increases the visual interpretability of an image by increasing the separation between different physical features in the image. These noises on images must be removed before applying image processing algorithms because these noises and unwanted information on the digital image affect the results to be obtained from the segmentation and feature extraction steps. In our project, these noises have been removed using Gaussian blur filtering techniques. Gaussian filter, a low-pass filter, results in a much clearer image than the original.

In general, image smoothing algorithms blur image details. Therefore, image sharpening filters should be applied to the image in addition to the smoothing filters. Sharpening filters are performed on images by means of digital differentiation. Laplacian filter is used in this step. It is an image sharpening filtering method that calculates the second derivatives of images and measures the rate at which the first derivatives change. In addition, image enhancement processes were applied. 

The images of the data set were scaled to 244 by 244 and normalized to (0, 1) values.

### Transfer Learning

Transfer learning is a deep learning method that uses a previously trained model with a large dataset in a similar task for a problem. Hardware and time savings can be achieved by transferring the weight values obtained from deep learning models pretrained with transfer learning to the network of different data sets. It can be quite difficult to obtain data and design models for different image processing problems. The problem-appropriate transfer learning method makes it possible to obtain higher performance with less data and saves time for the model designer. The solution to a similar problem can be faster and easier with transfer learning by transferring all or some of what the machine learning model has learned. 

#### VGG19 Transfer Learning

VGGNet is a simple and effective convolutional neural network model. The most important feature that distinguishes VGGNet from other models is the use of a pooling layer after binary or triple convolution layers. Two different model architectures are used, namely VGG-16 and VGG-19. A total of 41 layers are used in the VGG-16 model including the convolution layer, pooling layer, activation function, dropout layer, fully connected layer and classification layers. The VGG-19 model is created by adding 3 convolution layers in addition to the VGG-16 model. In this model, as in other models, the depth value of the matrices increases from the input layer to the output layer, while the height and width dimensions decrease. In addition, in every convolution operation, the input and output dimensions are the same, and a filter size of 3 × 3 and a 2-step filter size of 2 × 2 are used in the pooling layers. 

**Figure 3.**
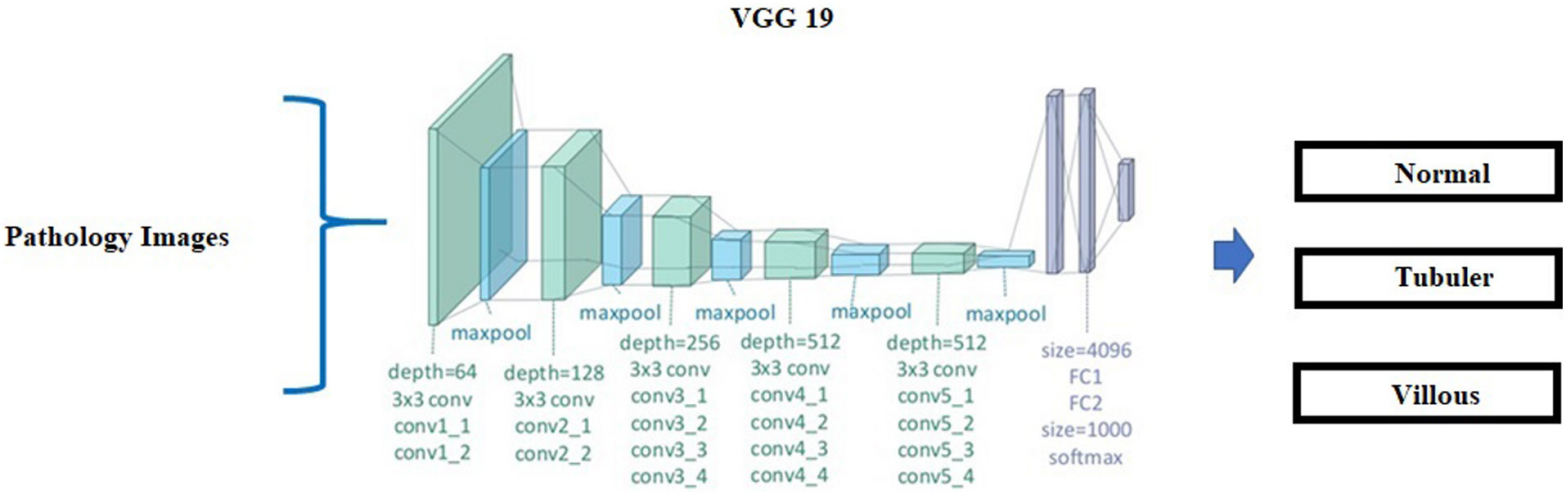
VGG-19 model.

In this project, the pretrained VGG-19 model will be used as we cannot use the large data set. Figure 3 shows the VGG-19 model of the proposed system. VGG is a CNN model used to classify images. The VGG-19 is useful for its simplicity as 3 × 3 folded layers are mounted on top, increasing with depth level. This architecture basically consists of 3 types of layers. The convolution layer is used to extract the feature from the image using different numbers and types of filters. The maximum pooling layer is used to reduce the image size and extract features from the feature map obtained from these filters in the convolution layer. The last layer is the maximum soft layer that estimates the probabilities of each class.^18^

#### DenseNet201 Transfer Learning

DenseNet201, one of the convolutional neural network models, is densely connected convolutional networks propounded by Huang et al.^19^ In the DenseNet architecture, information from earlier layers is combined with later layers rather than aggregated. This method provides advantages such as reuse of features and reducing the gradient disappearance problem. The most important architectural difference of DenseNet models from other models is that each layer in a dense block (except the first layers) receives additional inputs from all layers in that dense block and transmits its own feature maps to the next layers.

DenseNet201 contains 201 layers in its architecture. These 201 layers consist of 117 loops, 3 passes, and 1 classification layer.^20^ In the architecture of DenseNet models, each layer uses the properties of all previous layers as input. It can also give its own properties as input to the layers that come after it.^21^ The architecture that makes DenseNet architecture stand out is that it enables feature reuse by providing feature encrease. This situation also reduces the number of parameters.^19^ It consists of sequential stack normalization, ReLU and convolution operations. There are 7 × 7 convolution blocks and maximum pooling layer at the network entrance. After each density block comes the transition block. There are 1 × 1 convolutional layer, 2 × 2 average pooling layer and 4 density and transition blocks in the transition block. Finally, there is a 7 × 7 average pooling layer in the classification layer.

#### EfficientNetB7 Transfer Learning

As CNN architectures become more complex, the overall performance of the models tends to improve. There are 8 different EfficientNet models from 0 to 7. As the number of models increases, the number of parameters increases slightly, and the accuracy increases considerably. Unlike other CNNs, EfficientNet reduces the depth, width, and resolution of the network while reducing model complexity. Generally, it contains 7 blocks, where each block can contain an extra number of subblocks, and the number of these blocks increases from EfficientNetB0 to EfficientNetB7. The EfficientNetB0 model includes 237 layers and EfficientNetB7 includes 813 layers.

### Ensemble Learning

Ensemble learning is combining several models to eventually get a stronger model. There are different strategies for building ensemble models, including boosting, bagging, and stacking.

In our project, a coupling community model of 3 architectures (VGG19, ResNet50 and EfficientNetB7) was created. After the feature extraction layers of these models, the assembling process was performed. In other words, the features extracted by these models are combined into a single feature vector and then fed into fully connected dense layers to perform the classification task.

The overall architecture of the propounded ensemble model includes the simultaneous extraction of features from the 3 CNN architectures. These architectures take input images at 224 × 224 pixels. The outputs of the convolutional layers are passed through the max pooling and dense layers before assembling and form the feature vector. The outputs from these feature extraction submodels are smoothed and combined into a single long vector. After combining these 3 feature vectors, a fully connected interpretation layer is passed. Then, 2 dense layers and a dropout layer were added to prevent overfitting and to prevent increase generalization.

### Evaluation Criteria

Experimental studies in this article were performed on a computer with Intel (R) Core (TM) i7 9750U CPU @ 2.60 GHz, 6 GB NVIDIA GeForce GTX 1660 Ti graphics card, and 16 GB primary memory. The software was implemented using Python 3.7 and related libraries. 

At this stage, the performance of deep learning techniques will be made with different evaluation criteria. The confusion matrix will be used to evaluate the models created with classification algorithms and to determine which classification model produces more accurate results. The confusion matrix is a tabular layout developed to visualize the performance of a classifier. In this matrix, true positive (TP), false-negative (FN), false-positive (FP), and true negative (TN) numbers are given. Model performance will be evaluated by obtaining accuracy, sensitivity, recall, specificity, precision, and F1-score values using this matrix.^22^

## Results

Within the scope of this study, an artificial intelligence-based algorithm with a high accuracy rate that can be used in clinical pathology has been created for the diagnosis of normal colon epithelium and colon polyps (tubular adenoma and villous adenoma).

**Figure 4.**
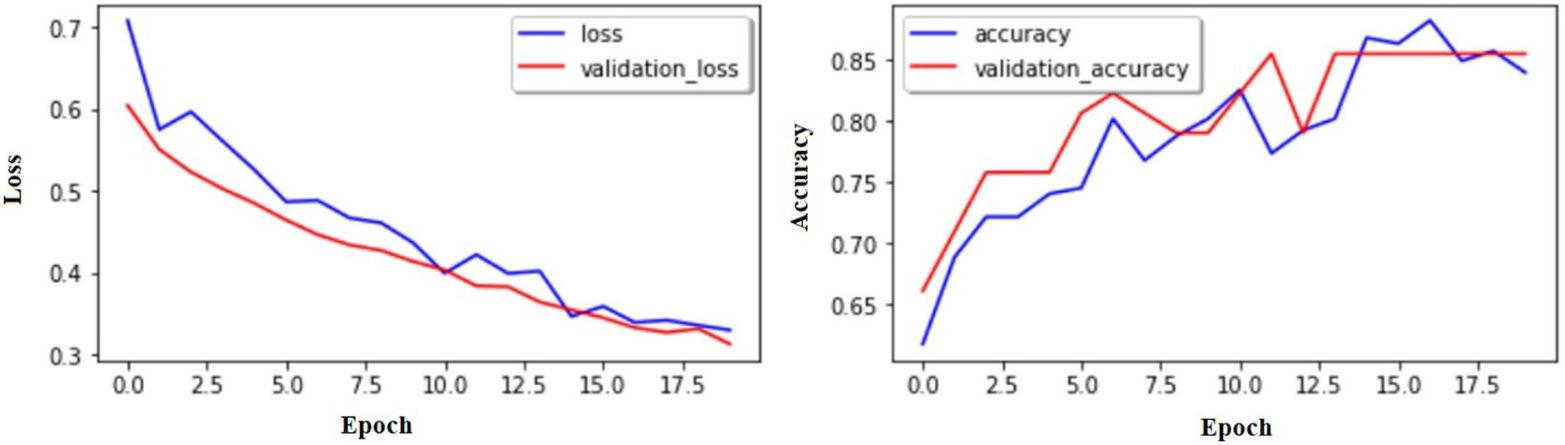
VGG-19 loss and accuracy results.

The results of the VGG-19 algorithm loss and accuracy values are as seen in Figure 4. As seen in Figure 4, the validation accuracy value was 85.48%. In the loss values, the validation loss value was obtained as 0.3136.

**Figure 5.**
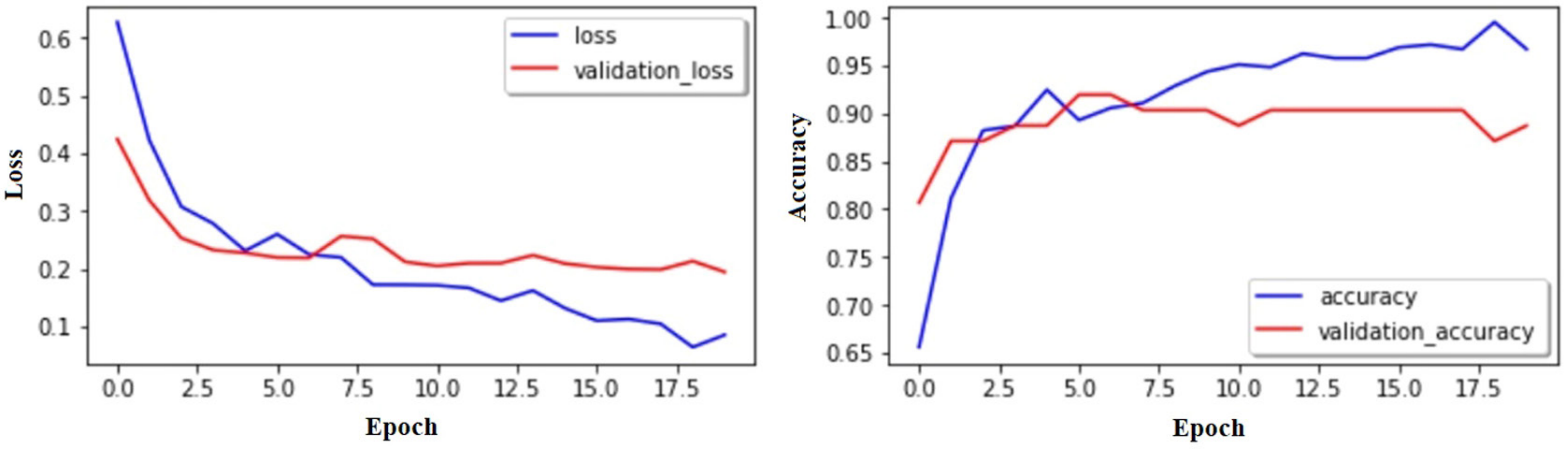
DenseNet201 loss and accuracy results.

The results of the DenseNet201 algorithm’s loss and accuracy values are as seen in Figure 5. As seen in Figure 5, the validation accuracy value was 88.71%. In the loss values, the validation loss value was obtained as 0.1946.

**Figure 6.**
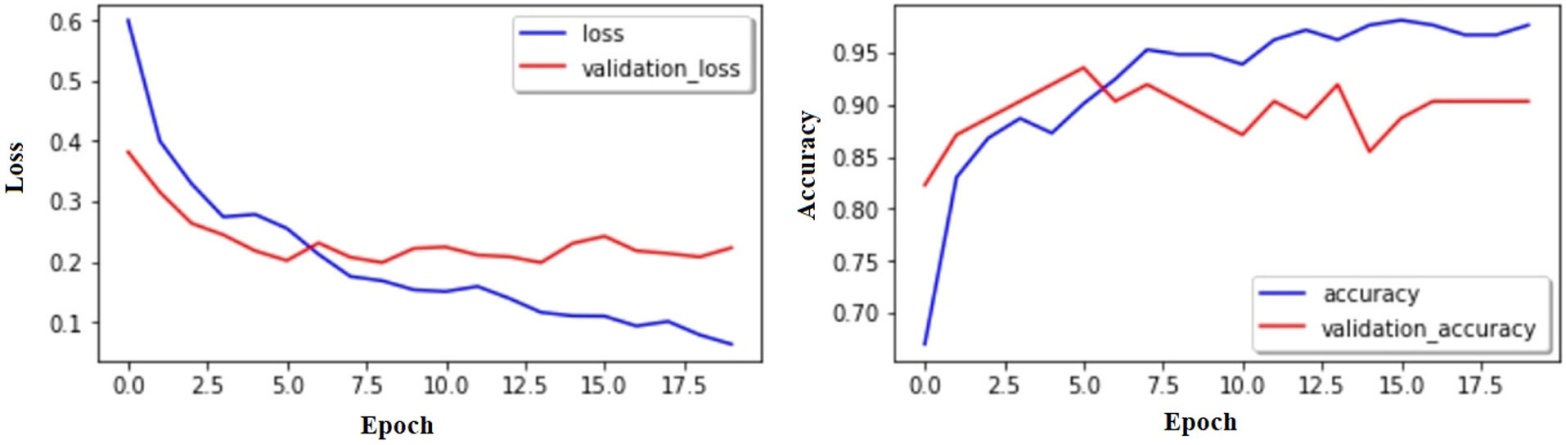
EfficientNetB7 loss and accuracy results.

The results of the loss and accuracy values of the EfficientNetB7 algorithm are as seen in Figure 6. As seen in Figure 6, the validation accuracy value was 90.32%. In the loss values, the validation loss value was obtained as 0.2229.

**Figure 7.**
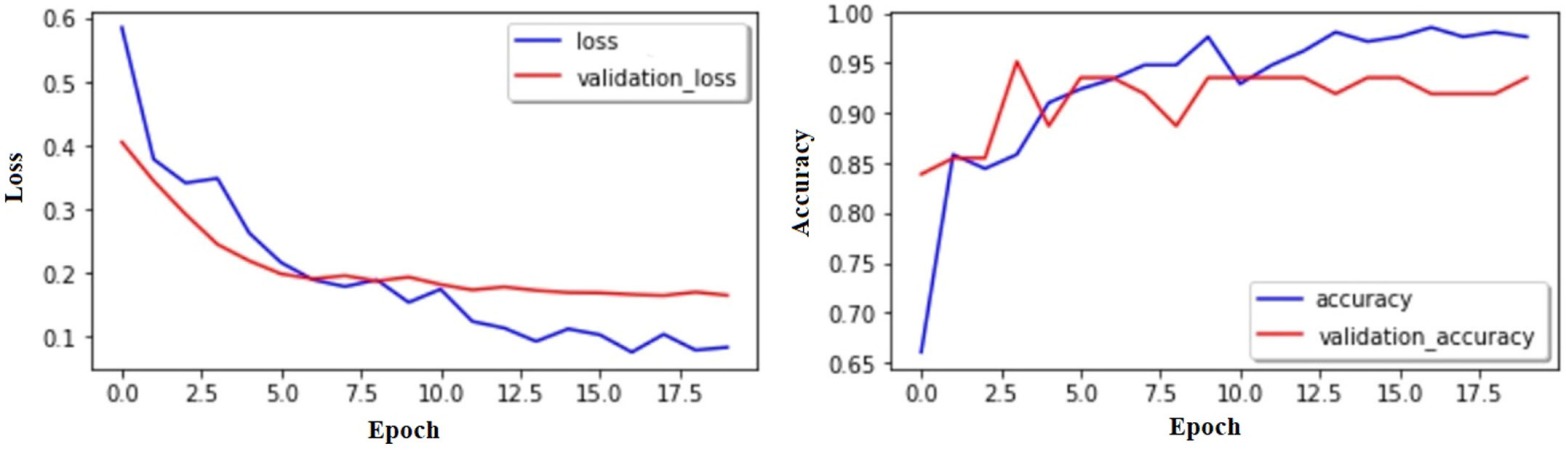
Ensemble learning loss and accuracy results.

In this study, a coupling ensemble model of 3 CNN architectures (VGG19, ResNet50, and EfficientNetB7) was created. The results of the loss and accuracy values of this model are as seen in Figure 7. As seen in Figure 7, the validation accuracy value was 94.17%. In the loss values, the validation loss value was obtained as 0.1653.

**Figure 8.**
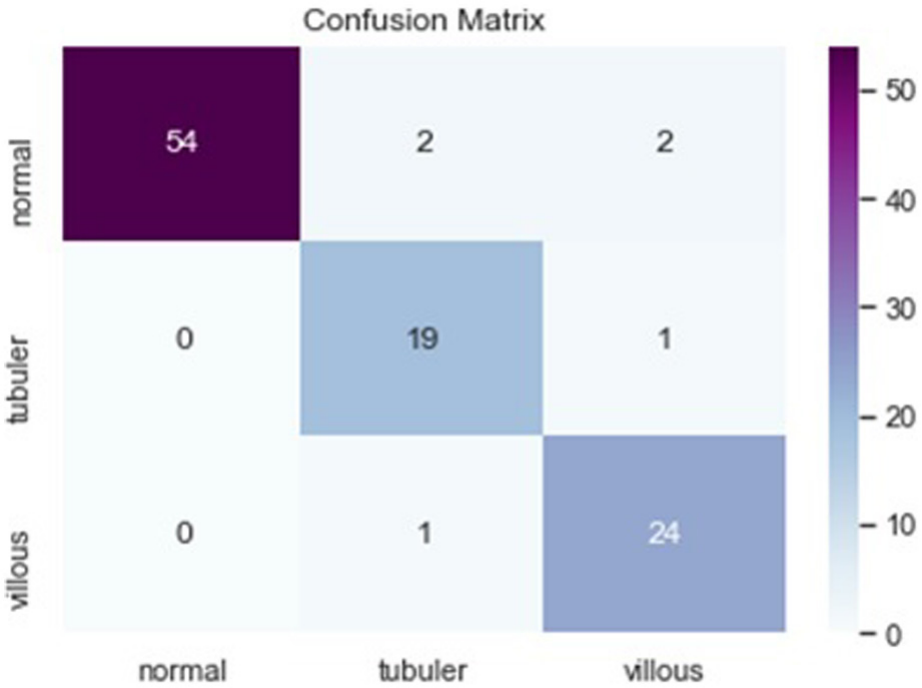
Ensemble learning confusion matrix.

In this project, 94.17% accuracy was achieved by using the ensemble learning approach. The resulting confusion matrix is seen in Figure 8. As it can be seen from Figure 4, samples in the normal class and 1 sample in the tubular and villous classes were misclassified.

According to the results obtained in our test data, 96.12% accuracy, 0.93 precision, and 1.0 recall were obtained in the normal group. The values obtained for the 3 classes are seen in Table 1. As seen in Table 1, the best F1-score value was obtained in the normal class.

According to the results of our test data, 94.17% accuracy, 95.12% sensitivity, and 90.48% specificity were achieved in the ensemble learning model. The results obtained from the algorithms are shown in Table 2. As seen in Table 2, the best accuracy and specificity values were obtained in the model that we recommend. This model works in 13 minutes and 34 seconds.

## Discussion

The large number of preparation samples that are sent from the clinical departments to the medical pathology department prolongs the time of diagnosis and delays the treatment to be applied. In addition, it has been shown that medical errors are associated with a qualitative and quantitative low number of personnel.^23^ In another study, it was shown that insufficient education, lack of support in decision-making and wrong decisions are some of the causes of medical errors.^24^ According to the minimum standards determined by the European Union of Medical Specialists Physicians (UEMS) Pathology Qualification Board, at least 7500 pathology materials must be examined during the education life of a student who will become a specialist.^25^ Delayed diagnosis of cancer diseases increases the morbidity and mortality rate. For this reason, it is vital to quickly examine and evaluate the preparations that come to pathology. In the literature, artificial intelligence-assisted diagnosis has been tried to be made using clinical and histopathological findings.^2^ In this study, although a path compatible with the literature was followed, a software with a higher accuracy rate than other artificial intelligence-supported studies was developed that recognizes different histomorphological images. For the study, an accuracy rate of 94.17% was achieved with 515 images at decimal augmentation.

While the average performance of some of the studies shown in Table 3 is 86.6%, this study achieved an accuracy rate of 94.17%. The 10% margin of error is caused by the lack of data (images obtained from the preparations), some of the data containing noise and the recognition capacity of the artificial intelligence algorithm. There are a limited number of studies in the literature on reading pathology images, which is also the subject of this study. Sena et al^26^ used a total of 393 colon preparation images divided into 4 categories (normal mucosa, paraneoplastic lesion, adenoma, and colon cancer) and achieved an accuracy value of 95%.

The most important advantage of our study compared to other studies in the literature is that the accuracy rate of the study is high compared to the data size. As stated in Table 3, achieving an accuracy rate of 94.17% with a data size of 515 makes this study stand out. The main reason for this is that 3 methods were used together. In addition, these advantages of the study may indirectly contribute to assistant training. Since most of the studies in the literature consist of colonoscopic videos or images, pathological studies have remained in the background. This is one of the reasons why this study is at a disadvantage. Namely, pathology preparation visuals are difficult for artificial intelligence to understand. This may differ between browsers. It has been shown that the brightness, contrast and sharpness of the preparation image affect the prediction performance.^27^ However, this study aimed at recognizing colon polyps has now created a method that can include many other diseases. The biggest advantage of this is that it supports that newly added diseases will have a high accuracy rate with the machine learning model used.

In this study, the hyperparameters of the deep learning model are determined by Grid Search method. Hyperparameters can significantly affect the success of the model. Correct hyperparameter settings are used for optimizing the training process, protecting against overfitting and achieving better results. In our project, the Grid Search method was used to find the best hyperparameter combination by trying certain values within a certain range of hyperparameters.

The study was designed with a total of 515 preparations under 3 data types. It was observed that the normal epithelium of the colon and colonic polyp (tubular adenoma and villous adenoma) preparations were recognized by artificial intelligence in 13 minutes and 34 seconds with an accuracy of 94.17%. In future studies, a web application will be developed and the model trained here will be run on the application. Thus, when an image is uploaded, its class will be determined immediately and time loss may be prevented drastically. In addition, by increasing the data set, new features will be tried on hybrid models and the success rate will be tried to be increased. This study will be a pioneer in the studies to be done in this field.

## Figures and Tables

**Table 1.** Evaluation Criteria

Class	Accuracy	Precision	Recall	F1 Score
Normal	96.12%	0.93	1.0	0.96
Tubular	96.12%	0.95	0.86	0.90
Villous	96.12%	0.96	0.89	0.92

**Table 2.** Results for Models

Algorithm	Accuracy	Specificity	Sensitivity	Time
VGG-19	0.8548	0.6667	0.9512	9 minutes and 23 seconds
DenseNet201	0.8871	0.8780	0.9048	10 minutes and 2 seconds
EfficientNetB7	0.9032	0.8571	0.9268	12 minutes and 39 seconds
Ensemble Learning	0.9417	0.9320	0.9320	13 minutes and 34 seconds

**Table 3.** Sample Studies Table

Study	Method	Data Size	Performance
Zuhang et al (2018)	ResYOLO	17 574	%88.6%
Tomita et al (2019)	ResNet-18	379	86.5%
Teramato et al (2017)	DCNN	298	71.1%
Korbar et al (2017)	ResNet	2074	93%
Wei et al (2020)	ResNet	1182	93.5%
Nasir et al (2021)	ResNet-18	1500	87%
Our study	VGG19+ DenseNet201+ EfficientNetB7	515	94.17%
